# The Remarkable Role of Triosephosphate Isomerase in Diabetes Pathophysiology

**DOI:** 10.3390/ijms26188809

**Published:** 2025-09-10

**Authors:** Mónica Rodríguez-Bolaños, Ruy Perez-Montfort

**Affiliations:** Departamento de Bioquímica y Biología Estructural, Instituto de Fisiología Celular, Universidad Nacional Autónoma de Mexico, Circuito Exterior S/N, Cuidad Universitaria, Mexico City 04510, CDMX, Mexico

**Keywords:** triosephosphate isomerase, diabetes, microRNAs, oxidative stress, cell membrane fluidity, ATP-sensitive channel, rapamycin

## Abstract

This work reviews the complex role of the enzyme triosephosphate isomerase (TIM) (EC 5.3.1.1) within the context of diabetes, a prevalent metabolic disorder. It summarizes the main biochemical pathways, cellular mechanisms, and molecular interactions that highlight both the function of TIM and its implications in diabetes pathophysiology, particularly focusing on its regulatory role in glucose metabolism and insulin secretion. TIM’s involvement is detailed from its enzymatic action in glycolysis, influencing the equilibrium between dihydroxyacetone phosphate and glyceraldehyde-3-phosphate, to its broader implications in cellular metabolic processes. The article highlights how mutations in TIM can lead to metabolic inefficiencies that exacerbate diabetic conditions. It discusses the interaction of TIM with various cellular pathways, including its role in the ATP-sensitive potassium channels in pancreatic beta cells, which are crucial for insulin release. Moreover, we indicate the impact of oxidative stress in diabetes, noting how TIM is affected by reactive oxygen species, which can disrupt normal cellular functions and insulin signaling. The enzyme’s function is also tied to broader cellular and systemic processes, such as membrane fluidity and cellular signaling pathways, including the mammalian target of rapamycin, which are critical in the pathogenesis of diabetes and its complications. This review emphasizes the dual role of TIM in normal physiological and pathological states, suggesting that targeting TIM-related pathways could offer novel therapeutic strategies for managing diabetes. It encourages an integrated approach to understanding and treating diabetes, considering the multifaceted roles of biochemical players such as TIM that bridge metabolic, oxidative, and regulatory functions within the body.

## 1. Introduction

Diabetes is a chronic metabolic disorder characterized by high incidence and increasing prevalence in recent years [1,2,3,4,5]. This condition has numerous severe complications and leads to the emergence of various comorbidities [1]. According to the current classification by the World Health Organization, diabetes has been categorized into two primary types: type 1 and type 2 [6]. However, other forms, such as gestational diabetes, have been described, and some authors now propose the existence of a new type, which has been denominated as type 3 [7,8,9].

Type 1 diabetes, representing 5–10% of reported cases [1,10], is characterized by a lack of insulin secretion due to damage to pancreatic beta cells, typically induced by an autoimmune response. Type 2 diabetes accounts for approximately 90% of cases, and the associated pathological mechanism involves the accumulation of fat in skeletal muscle and the liver, generating resistance to insulin action in these tissues. In compensation for this increased demand, pancreatic beta cells augment insulin secretion until dysfunction of the insulin-producing cells occurs [1,3]. Years before diagnosis, dysfunction in glucose regulation has been identified in various tissues that control glucose homeostasis [3]. Predisposing factors for the onset of the disease include genetic factors, alteration of fat storage sites—which explains why individuals with adequate weight can develop type 2 diabetes—obesity, sleep cycle disorders, circadian rhythm abnormalities, and insufficient physical activity [1].

Gestational diabetes develops during the later stages of pregnancy as a result of hormones secreted by the placenta (placental human lactogen, progesterone, cortisol, prolactin, and growth hormone) to promote fetal growth [11]. Due to metabolic changes during this period, women develop insulin resistance, which is exacerbated as pregnancy advances due to the action of the aforementioned hormones, until, ultimately, the patient’s metabolic state declines and carbohydrate intolerance ensues [1].

A connection between diabetes and a variety of mental illnesses has recently been demonstrated, with observations indicating that patients with diabetes exhibit a 1.5 to 2 times higher probability than non-diabetic patients of experiencing cognitive decline [1,5]. A correlation between the development of Alzheimer’s disease and type 2 diabetes has also been described [3,4,5,12]. Moreover, in a mouse model with Alzheimer’s and type 1 diabetes, increased accumulation of beta-amyloid peptide and tau phosphorylation has been observed in the brains of these individuals [13].

While the cognitive dysfunction associated with diabetes is multifactorial and the related neurological mechanisms remain unknown, evidence from preclinical and clinical trials indicates that insulin resistance, imbalance of the redox system, failure of cerebral microvascular function, alterations in the balance of metals and ions, and dysfunction in the brain’s drainage system contribute to its development [5]. Insulin, a key protein in maintaining cellular homeostasis, was initially thought to be produced exclusively by the beta cells of the islets of Langerhans; however, certain types of cells in the brain have also been found to synthesize insulin. In the brain, insulin helps diminish inflammatory responses, and the binding of insulin to its receptor can translocate this complex to the nucleus, where it functions as a transcription factor for genes associated with diseases such as cancer, diabetes, Huntington’s disease, Alzheimer’s disease, and Parkinson’s disease [14].

The term “type 3 diabetes” results from a conjunction of the pathological mechanisms observed in diabetes and Alzheimer’s disease, primarily insulin resistance, persistent hyperglycemia, and cognitive deficits. Neuronal apoptosis is provoked by hyperglycemia and insulin resistance, which also favor the formation of beta-amyloid peptide deposits. Under normal conditions, excess beta-amyloid is eliminated by lipoprotein receptor 1, whose expression decreases in the presence of insulin resistance or during degradation processes involving the insulin-degrading enzyme. Chronic reduction in insulin leads to a decrease in its concentration in the brain, directly affecting functions such as learning, long-term memory, expression of proteins such as acetylcholinesterase, and reduction in the phosphorylation of tau protein [8].

Hyperglycemia and diabetes impair the function of various proteins in the organism, including triosephosphate isomerase (TIM), whose expression and activity are compromised. Structural modifications of TIM, driven by oxidative and nitrative stress, lead to the accumulation of methylglyoxal (MG), a precursor of advanced glycation end products (AGEs). MG promotes further oxidative stress and structural alterations of biomolecules, exacerbating metabolic dysfunction.

Understanding the link between diabetes and TIM highlights TIM as a potential therapeutic target to disrupt diabetic complications and improve glucose metabolism.

## 2. Triosephosphate Isomerase

**Figure 1:**
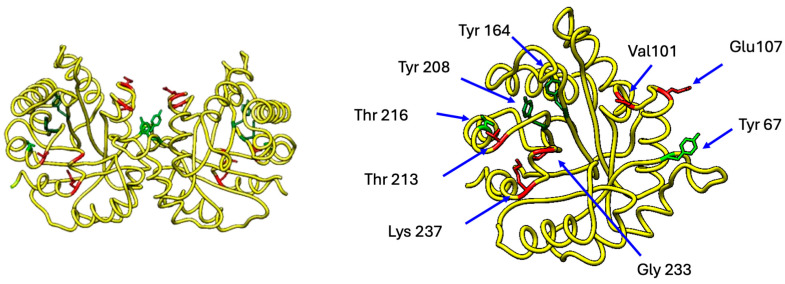
Structure of human triosephosphate isomerase showing some selected relevant residues. The figure shows two perpendicular views of the protein. A dimer of TIM is shown on the left panel, and a monomer rotated 90 degrees is shown on the right panel, displaying some of the residues involved in diabetes pathophysiology. The residues highlighted in red are those involved in the interaction with the Kir6 and SUR1 subunits of the K ATP channels. Residues in green are those that can be modified by the action of reactive nitrogen species (RNS). Molecular graphics and analyses were performed with UCSF Chimera, Version 1.19, developed by the Resource for Biocomputing, Visualization, and Informatics at the University of California, San Francisco using the Protein Data Bank Structure 4POC.

The enzyme triosephosphate isomerase (TIM) (EC 5.3.1.1), also known as TPI, TPID, or HEL-S-49, is a ubiquitous protein of approximately 250 amino acids per monomer, although the number of residues may vary depending on the species. It does not require any cofactors, and its function is solely limited by the diffusion of the substrate to the active site [15,16,17]. Triosephosphate isomerase (TIM) catalyzes the interconversion of glyceraldehyde 3-phosphate (G3P) and dihydroxyacetone phosphate (DHAP). G3P is converted to pyruvate, yielding two molecules of ATP and one NADH per G3P molecule. Efficient ATP and NADH production from glucose requires rapid DHAP-to-G3P conversion. TIM comprises two identical (β/α)8-barrel subunits, with catalytic sites located at the C-terminal ends. The TIM barrel motif is among the most common protein folds (~10% of proteins possess it; Figure 1). A loop following β-strand 6 closes over the active site, shielding substrates from solvent.

TIM’s catalytic mechanism involves proton transfer from DHAP C1 to Glu165, then to C2 of the *cis*-enediol intermediate to form G3P. This competes with solvent proton exchange via protonated Glu165. The enediol intermediate is stabilized by hydrogen bonds with the neutral imidazole of His95 and the ε-ammonium group of Lys13. A planar conformation enforced between inorganic phosphate and the enediol prevents premature elimination. The detailed mechanism is described by Knowles, Wierenga, and Helliwell [16,17,18,19,20]. TIM is exclusively active as a dimer despite each monomer containing a catalytic site [16,17,18,19,20].

**Figure 2:**
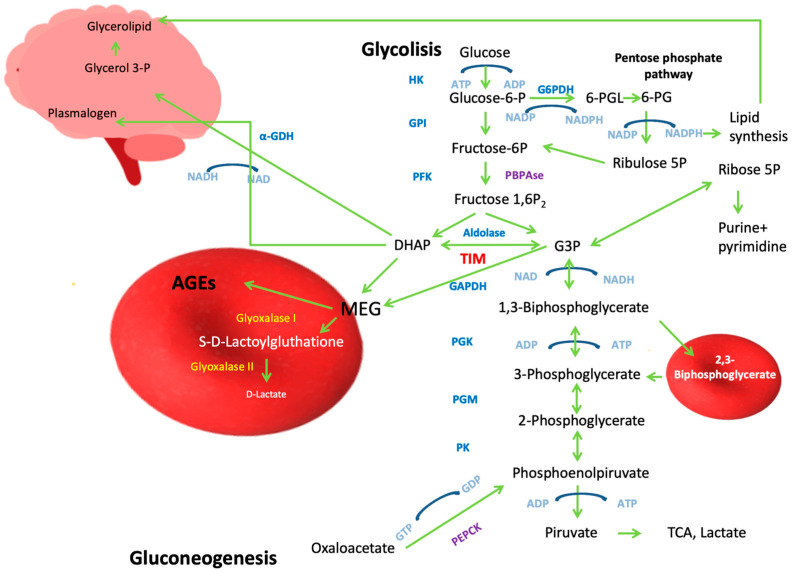
TIM and interconnected metabolic pathways (modified from Orosz, 2009 [23]). Glycolysis and the pentose phosphate pathway interconnect via fructose 6-phosphate and glyceraldehyde 3-phosphate. The pentose phosphate pathway generates NADPH, driving lipid synthesis. In the brain, DHAP is utilized for lipid synthesis through α-glycerophosphate dehydrogenase (α-GDH), which converts DHAP to glycerol-3-phosphate—a substrate for phospholipid and triacylglycerol synthesis. Under low NADPH conditions, DHAP is diverted to plasmalogen synthesis, while in erythrocytes, DHAP converts to methylglyoxal (MEG), yielding advanced glycation end products (AGEs). Gluconeogenesis synthesizes glucose from precursors such as lactate (via the Krebs cycle, where mitochondrial oxaloacetate is exported and converted to phosphoenolpyruvate (PEP)) or from DHAP (which accumulates during TIM deficiency—a critical enzyme in this process—or derives from adipose tissue glycerol and triacylglycerols). Impaired TIM activity blocks DHAP-to-G3P interconversion, inhibiting glycerol-derived gluconeogenesis and promoting DHAP conversion to MEG. Abbreviations used in Figure 2: BPGM, Bisphosphoglycerate mutase; BPGP, Bisphosphoglycerate phosphatase; ENO1, Enolase; G6PDH, Glucose-6-phosphate dehydrogenase; GPI, Glucose-6-phosphate isomerase; GSH, Glutathione; GAPDH, Glyceraldehyde-3-phosphate dehydrogenase; GDH, Glycerol-3-phosphate dehydrogenase; HK, Hexokinase; O.P., Oxidative phosphorylation; PFK, Phosphofructokinase; PGK, Phosphoglycerate kinase; PGM, Phosphoglycerate mutase; PK, Pyruvate kinase; TCA, Tricarboxylic acid cycle; PEPCK, Phosphoenolpyruvate carboxykinase; PBPase, Fructose-1,6-bisphosphatase.

These metabolites, in turn, interconnect with other metabolic pathways such as the pentose phosphate pathway, gluconeogenesis, and fatty acid synthesis (Figure 2) [21,22,23]. In humans, there is a single gene encoding this enzyme located on chromosome 12p13.31, and, to date, this locus is known to encode only a unique form, as no isoforms resulting from alternative messenger RNA processing (alternative splicing) have been identified. However, a wide range of polymorphisms or mutations have been reported [24,25,26,27,28,29,30,31,32,33,34,35,36,37,38,39]. These mutations generate enzymes with very particular characteristics in terms of their enzymatic activity and stability, causing diseases such as triosephosphate isomerase (TIM) deficiency—a rare autosomal recessive disorder with an allele carrier frequency of 0.4–1% in Caucasians/Asians and 4% in African Americans. Most patients exhibit hemolytic anemia manifesting as jaundice, alongside frequent reticulocytosis and hyperbilirubinemia. Recurrent bacterial infections occur, predominantly respiratory. Progressive neurological dysfunction emerges at 6–24 months, featuring dystonia, tremors, dyskinesia, cardiomyopathy, spinal motor neuron impairment, and progressive muscle weakness. Cognitive function remains intact or mildly affected; developmental delays primarily stem from motor dysfunction impacting mobility and speech. Mortality typically occurs before age 6.

The Glu104Asp substitution accounts for 80% of cases. Other mutations—primarily compound heterozygous with E104D—modify disease severity: Ile170Val and Phe240Leu suppress neurological degeneration, while Cys41Tyr and Val231Met delay its onset. Only ~100 cases are reported due to extreme rarity. Animal models recapitulating human phenotypes are recent; *Drosophila melanogaster* studies indicate dimer stability (rather than catalytic function) dictates symptom severity. No curative treatment exists; management relies on packed red blood cell transfusions, palliative care, and respiratory support as needed.

The low activity of TIM in patients with this disease causes an imbalance in glycolytic efficiency in their tissues, starting with the accumulation of DHAP. As previously mentioned, TIM expression occurs in all tissues; however, some possess higher expression levels of this enzyme and, consequently, greater glycolytic capacity [39].

Functionally, TIM undergoes a series of post-translational modifications that alter its properties to allow regulation of its activity, enabling adaptation to the cell’s metabolic needs. These modifications include phosphorylation, acetylation, glycosylation, ubiquitination, methylation, deamidation, nitration, dopaminylation, nitrosylation, and nitrotyrosination [39,40,41,42]. Recently, new functions have been described for TIM in addition to its well-known catalytic activity [43,44] (Figure 1). These moonlighting functions broaden our understanding of the roles of this protein, prompting the exploration of its role not only in conditions related to its enzymatic activity but also in its moonlighting activities. For this reason, in this review, we will examine the possible role of this enzyme in the development of diabetes.

## 3. MicroRNAs—miR-193b-3p, miR-1285-3p, and TIM

MicroRNAs (miRNAs) are small non-coding RNA molecules (20–22 nucleotides in length) responsible for RNA gene silencing and post-transcriptional regulation of gene expression (degradation) through binding to the 3′ untranslated region (3′ UTR) of mRNA. This binding permits the regulation of protein translation quantities and, consequently, the control of numerous cellular, biological, and metabolic processes. miRNAs are produced in various tissues and can be actively distributed by their release into the bloodstream within exosomes [44]. Aberrant expression of miRNAs can lead to the under- or overexpression of key enzymes involved in the development of diseases such as diabetes itself, thereby contributing to the disease’s etiology [3,14,45,46]. Multiple studies have focused on elucidating the relationship between miRNAs and glucose metabolism; for instance, miR-122, miR-126, and miR-20b-5p affect insulin function and are elevated in patients with type 2 diabetes [45]. The search for miRNAs that can function as biomarkers of diabetes aims to monitor disease progression and enable early detection to prevent its advancement.

**Figure 3:**
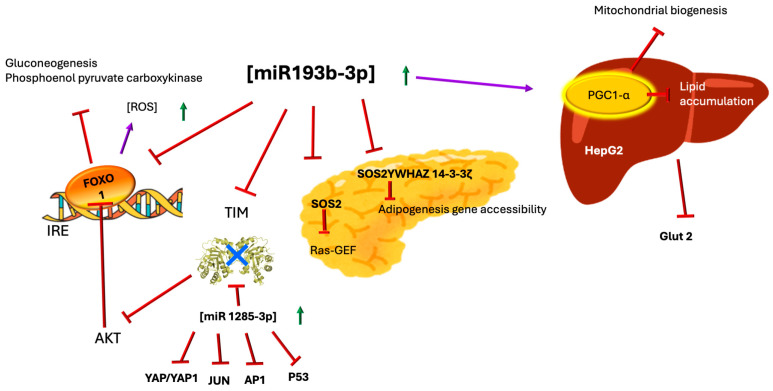
Effect of miRNAs on TIM expression. When the expression of miR193b-3p increases, the expression of several proteins related to carbohydrate metabolism in various organs (e.g., pancreas, the liver) is repressed. Among these is TIM, whose lack of activity affects inositol expression, thereby impacting the signaling of several pathways, such as the AKT pathway (see Figure 4, below), which, in turn, affects the expression of other proteins involved in glycogenesis. Additionally, this facilitates the formation of reactive oxygen species (ROSs). PGC-1α not only regulates lipid accumulation but also governs mitochondrial biogenesis and energy metabolism. Additionally, miR193b-3p targets GLUT2, a glucose transporter whose downregulation exacerbates hyperglycemia. On the other hand, miR-1285-3p also inhibits the expression of several proteins related to growth, differentiation, and tumor suppression, as well as some associated with carbohydrate metabolism, such as TIM and p53.

In this context, miR-193b-3p has been found to play a crucial role in glucose metabolism (Figure 3).

It has been observed that in mice with glucose intolerance, differential expression of miR-193b-3p occurs after exercise therapy, showing higher concentrations in rodents before the therapy [46]. Additionally, miR-193b-3p exhibits differential expression in patients with polycystic ovary syndrome accompanied by insulin resistance and glucose metabolism disorders when compared with patients with the same syndrome but normal glucose tolerance. Moreover, in the plasma of human patients with altered fasting glucose levels and those with glucose intolerance, the miRNA hsa-miR-193b-3p is overexpressed [46]. Elevated expression of miR-193b-3p is also observed in patients with recently diagnosed and untreated diabetes (two-fold increase). In vitro, miR-193b-3p can affect glucose metabolism by inhibiting the expression of SOS2 and YWHAZ/14-3-3ζ—regulators of beta-cell survival and adipogenesis—thereby deregulating the transcription factor FOXO1, which is downstream of the PI3K-AKT pathway, demonstrating that these miRNAs have a critical role in diabetes development [3]. Among the direct targets of miR-193b-3p are the gene and the protein of peroxisome proliferator receptor gamma coactivator 1 alpha (PPARGC1A and PGC-1α, respectively). PGC-1α also interacts with the nuclear receptor PPAR-γ, whose reduced expression leads to lipid accumulation in HepG2 cells and alterations in genes related to lipid processing. This results in a reduction in glycolysis and decreased mitochondrial activity because PGC-1α participates in mitochondrial biogenesis and regulates gluconeogenesis in the liver, redirecting metabolites toward lipogenesis [46]. Another direct target of miR-193b-3p is TPI1 mRNA; higher levels of miR-193b-3p correspond to approximately 60% decreased levels of TIM in plasma. As previously mentioned, TIM is a key enzyme linking energy metabolism with redox metabolism. An imbalance in pro-oxidative/antioxidative homeostasis due to the absence of TIM activity increases the accumulation of oxidant species in patients with diabetes (see oxidative stress section below) [30]. Transcript analysis suggests that miR-193b-3p affects glucose metabolism by inhibiting the translation of proteins such as the insulin receptor and GLUT2, thus preventing glucose uptake [3,46]. Furthermore, in patients with Parkinson’s disease, deregulation of miR-193b-3p levels has been observed (the levels change as the disease progresses) which, in turn, regulates the expression of PPARGC1α. Deregulation of the miR-193b-3p/PPARGC1α pathway directly leads to alterations in insulin expression, underscoring the importance of insulin in Parkinson’s disease [14].

Conversely, miR-1285-3p is implicated in various types of cancer; for example, it is associated with the inhibition of migration in pancreatic and liver cancer cells [45]. Additionally, miR-1285-3p is considered a valuable biomarker for early diagnosis in patients with diabetes and pancreatic ductal adenocarcinoma—a lethal tumor—since its expression is differentially downregulated in patients with these pathologies compared with healthy individuals [47]. In patients with chronic heart failure, miR-1285-3p expression is increased by almost 132% [48].

Among the targets of miR-1285-3p are YAP/YAP1 (involved in the HIPPO signaling pathway related to cellular proliferation and migration in T3M4 and SU.8686 cell lines), Jun (a proto-oncogene product that activates the AP1 protein responsible for proliferation, cellular differentiation, apoptosis, and control of oncogenic transformation), and p53 (which participates in metabolism and diabetes development by promoting insulin resistance through regulation of the GLUT-1 receptor). In Sertoli cells of *Ovis aries*, where glycolytic activity is elevated, one of the principal targets of miR-1285-3p is TIM, since it binds to the 3′ UTR region of its gene, modulating its expression [49].

Glycolysis is critically important for male mammalian reproductive system development, particularly in testicular and epididymal maturation [48,49,50,51,52]. The *TPI* gene encoding TIM is expressed in sperm heads, and anti-TIM antibodies inhibit the acrosomal reaction essential for sperm–zona pellucida binding [53]. In bull sperm, TIM localizes to the acrosomal plasma membrane [54], while mouse studies report TIM overexpression in sperm flagella [55]. As oxidative phosphorylation powers sperm motility, TIM regulates the associated energy metabolism [54]. Aberrant glycolytic metabolism impedes spermatogenesis, causing male infertility [56]. The miR-1285-3p—overexpressed in diabetes—may suppress TIM translation, potentially compromising fertility. This mechanism is documented in *Ovis aries* but remains unexplored in humans. Diabetes-induced male infertility likely involves multiple factors, including oxidative stress and dysfunction of TIM.

Since TIM plays an important role in the development and maturation of the male reproductive system, failures in glycolysis can lead to infertility [53,57,58]. Although not confirmed in humans, the overexpression of miR-1285-3p in diabetes could contribute to infertility by inhibiting the expression of TIM; this hypothesis deserves to be investigated in the future.

## 4. Regulation of Insulin Secretion

**Figure 4:**
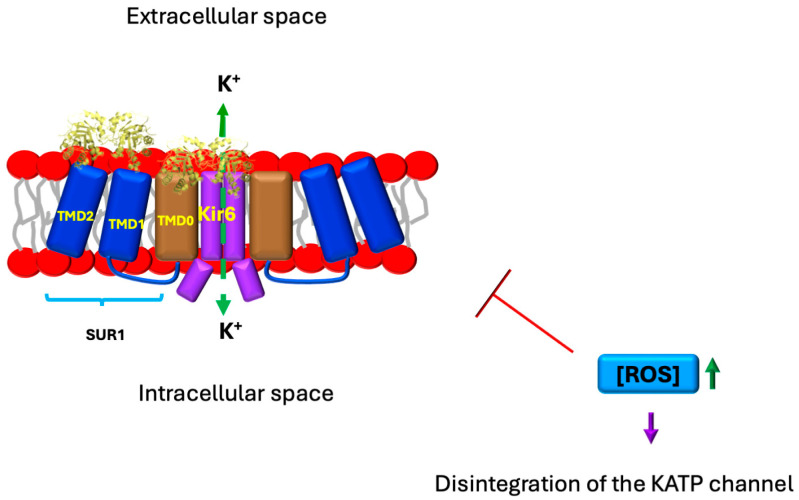
Interaction of TIM with K ATP. Based on the docking analysis performed by Daniel et al. [59], it was found that TIM interacts with TMD1 and TMD2 of SUR1 in K ATP on the extracellular side. TIM shows weak interactions with TMD0. In contrast, TIM interacts with all subunits of Kir6. The residues of TIM proposed to be involved in the interaction are Val 101, Glu 107, Thr 213, Gly 233, and Lys 237, which are located in the inner loops connecting the beta sheet to the alpha helix (see Figure 1). These interactions alter the mechanisms that regulate the opening or closing of the channel. One of the forms of regulation of K ATP channels is dependent on lipid raft integrity and reactive oxygen species (ROS) levels. Elevated ROS concentrations trigger lipid peroxidation in phospholipids, destabilizing raft architecture, promoting structural disintegration, and, thereby, impairing channel function.

ATP-binding cassette (ABC) transporters constitute a large superfamily of proteins responsible for transporting numerous molecules across membranes. ATP-sensitive potassium channels (K ATP) function as sensors of the intracellular ATP/ADP ratio that regulate the channel’s activity and affect the transport of potassium across the membrane (Figure 4).

These channels are composed of the pore-forming units Kir6.1 and Kir6.2, as well as the regulatory ABC transporters sulfonylurea receptor 1 and 2 (SUR1 and SUR2). In pancreatic beta cells, K ATP channels are responsible for coupling glucose metabolism with insulin secretion, and in this tissue, these receptors are mainly composed of SUR1 and Kir6.2. That is, this channel regulates insulin secretion in pancreatic beta cells; therefore, when mutations occur in the genes encoding SUR1 (ABCC8) and Kir6.2 (KCNJ11), serious problems in insulin secretion arise, leading to neonatal diabetes or hyperinsulinism [60].

In a study conducted by Daniel et al. (2021) [59] to investigate potential contact-independent interactions between endothelial cells and insulin-secreting beta cells in rats, a dose- and time-dependent attenuator of insulin secretion was found, acting in both phases of insulin secretion. This factor turned out to be TIM, and upon performing a docking simulation, it was observed that TIM could interact with the conserved TMD1 and TMD2 regions of SUR in the K ATP channel. It is believed that this interaction between TIM and SUR could generate allosteric changes that prevent the closing of the K ATP channel [59]. Previously, it was also demonstrated that TIM, along with other glycolytic enzymes—glyceraldehyde 3-phosphate dehydrogenase (GAPDH) and pyruvate kinase (PK)—associates with the Kir subunits of K ATP channels. All of these enzymes, upon binding to Kir, inhibit the channel-opening activity, even after glucose stimulation [61]. K ATP channels are metabolic sensors responsible for regulating hyperglycemia by controlling insulin secretion, making this regulation mediated by TIM highly novel.

**Figure 5:**
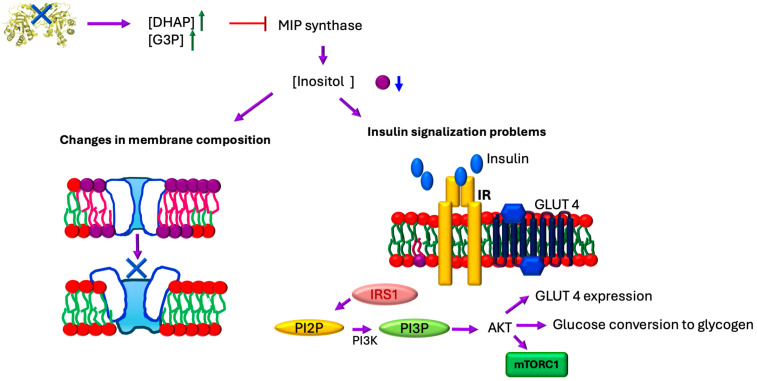
Effect of the lack of TIM activity. The lack of activity of TIM disrupts glycolysis, leading to the accumulation of DHAP and G3P, which inhibit de novo myoinositol biosynthesis by suppressing MIP synthase. This inositol deficiency alters phospholipid membrane composition, increasing viscosity and reducing plasticity, thereby impairing the proper localization and function of membrane-associated proteins. Additionally, the deficit of inositol phospholipid derivates (PIP2 and PIP3) disrupts critical signaling pathways, particularly insulin-mediated glucose metabolism. When insulin binds to the IRS-1 receptor, phosphorylation activates PI3K, which catalyzes PIP2 to produce PIP3; this second messenger then recruits AKT to regulate GLUT 4 translocation for glucose uptake, activating mTOR for anabolic processes and modulating glycogen synthesis. Thus, TIM dysfunction creates a cascade of effects.

In addition, the activity of TIM regulates the synthesis of inositol [44,62] (Figure 5).

The concentration of inositol is critical for insulin signaling as it participates through the formation of numerous second messengers that respond to the action of this hormone. Elevated glucose concentrations decrease the levels of absorption and biosynthesis of inositol and increase its excretion in urine (in patients with type 1 and type 2 diabetes). Insulin resistance, as well as increased glucose concentrations, reduce inositol levels in tissues [63].

Throughout this review, we describe how TIM exhibits changes in activity or expression in patients with diabetes, so this new regulation through this enzyme opens the door to understanding why insulin secretion occurs aberrantly in this disease.

## 5. TIM, Sororin, and the mTORC1 Pathway

**Figure 6:**
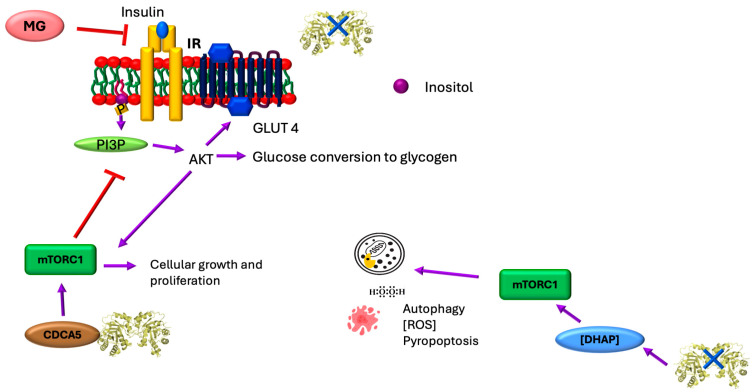
TIM and the mTOR pathway. In various pathological conditions, the presence or absence of TIM can activate the mTOR pathway. The first scenario is detailed in the upper left part of the figure, where the absence of TIM affects membrane composition and PI3P signaling. TIM has a moonlighting function in cancer by binding to CDCA5 (sororin, which is overexpressed in many cancer types). This interaction represses PI3P function, which is involved in autophagy signaling and vesicular trafficking. By inhibiting autophagy, cancer cells grow uncontrollably. The TIM-CDCA5 interaction also inhibits AMPK activity. Under normal metabolic conditions, AMPK suppresses mTORC1 activity [66,67]. This disruption impairs glucose metabolism but promotes cell growth and proliferation, contributing to metastasis. When TIM is inhibited or mutated, DHAP accumulates; this excess can activate mTORC1, which suppresses autophagy and promotes pyroptosis (inflammatory cell death) and ROS production. DHAP can influence mTORC1 activation through indirect metabolic signaling; abundant DHAP levels indicate abundant glucose availability, which cells interpret as a growth-permissive signal [68]. Under conditions of elevated oxidative stress, proteins such as TIM undergo post-translational modifications, leading to functional impairment. This dysfunction promotes the accumulation of toxic byproducts such as methylglyoxal (MG). MG impairs IRS-1 phosphorylation and PI3K/AKT signaling, which are critical for glucose transporter translocation, and it also disrupts insulin signaling cascades, further exacerbating metabolic dysregulation [69,70].

The mammalian target of rapamycin (mTOR) signaling pathway integrates intra- and extracellular signals to regulate metabolism, influencing diverse cellular processes including mitochondrial function, lipogenesis, ketogenesis, glucose metabolism, insulin signaling (and resistance), tumor development, angiogenesis, adipogenesis, and T lymphocyte activation [64,65]. Dysregulation of this pathway contributes to diseases such as type 2 diabetes and cancer [64]. mTOR, a 289 kDa highly conserved serine/threonine kinase, forms two multiprotein complexes—mTORC1 and mTORC2 [64]. mTORC1 regulates cell growth and proliferation while coordinating anabolic processes such as protein, lipid, and organelle synthesis; it also suppresses catabolic pathways such as autophagy [64] (Figure 6).

The mTORC1 and mTORC2 complexes exhibit an interesting functional dichotomy in diabetes pathogenesis [71]. While mTORC1 induces insulin resistance in adipose tissue, skeletal muscle, and liver through IRS-1 phosphorylation-mediated PI3K inhibition, it paradoxically enhances oxidative metabolism in skeletal muscle. Furthermore, mTORC1 serves dual roles in pancreatic β-cells; it stimulates insulin secretion while expanding β-cell mass and size, collectively lowering blood glucose. This explains why mTORC1 inhibitors frequently cause hyperglycemia as a primary side effect [71,72].

In cancer biology, TIM emerges as a significant regulator, with its dysregulation serving as a potential prognostic biomarker. In MGC-803 cells, TIM coordinates with sororin (CDCA5) to activate the PI3K/AKT/mTOR pathway—a critical relationship since TIM is essential for sororin expression, and sororin mediates sister chromatid separation during mitosis [38,66]. This TIM-mediated pathway activation drives oncogenesis through multiple mechanisms; mTORC1 stimulates proliferation, and AKT suppresses apoptosis while enhancing glycolysis (via GLUT4 membrane translocation and glycolytic enzyme activation), collectively promoting metastasis [38]. The physiological role of TIM in PI3K/AKT/mTOR regulation remains unclear, although DHAP activates this pathway. In diabetic kidney disease, DHAP accumulation triggers organ damage, particularly in renal podocytes and tubular cells, through the mTORC1/ROS/NLRP3 pyroptosis pathway (pyroptosis being an inflammatory form of programmed cell death; Figure 6). While the activation mechanism requires further elucidation, current evidence suggests that DHAP activates mTORC1 via the GATOR-Rag pathway, leading to ROS overproduction, impaired autophagy, and mitochondrial dysfunction. These changes stimulate proinflammatory molecule release and likely induce pyroptosis [70].

## 6. Oxidative Stress and TIM

**Figure 7:**
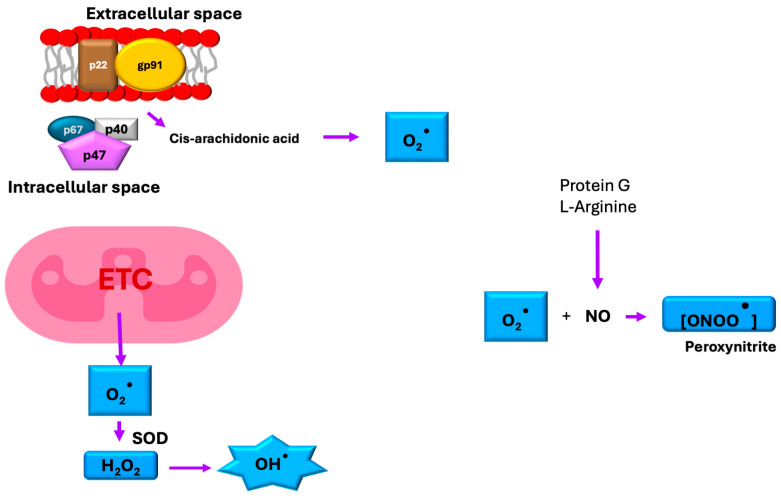
Reactive oxygen and nitrogen species. Reactive oxygen and nitrogen species are generated during basal metabolism. The primary source of ROS production is the electron transport chain (ETC) in the mitochondria, where superoxide dismutase (SOD) converts superoxide into hydrogen peroxide, which can subsequently give rise to a hydroxyl ion. Another significant source is NADPH oxidase, which uses arachidonic acid as a lipid mediator, leading to its activation and the generation of multiple ROSs. During the metabolism of L-arginine and through the action of G proteins, nitric oxide (NO) is produced, which interacts with other ROSs to form additional free radicals.

Oxidative stress occurs when cellular redox balance is disrupted, leading to damaging effects on cell membranes and biomolecules, including DNA, proteins, and lipids [4]. The primary reactive oxygen species (ROSs) involved include superoxide (O_2_^•−^), hydroperoxyl radical (HO_2_^•^), and the hydroxyl radical (^•^OH), and the primary reactive nitrogen species (RNS) include nitric oxide (^•^NO), and peroxynitrite (ONOO^−^). While mitochondrial electron transport serves as the major source of ROS generation, these reactive molecules also originate from other metabolic processes such as immune responses, arachidonic acid metabolism, and NADPH oxidase (NOX) enzyme activity [6] (Figure 7).

**Figure 8:**
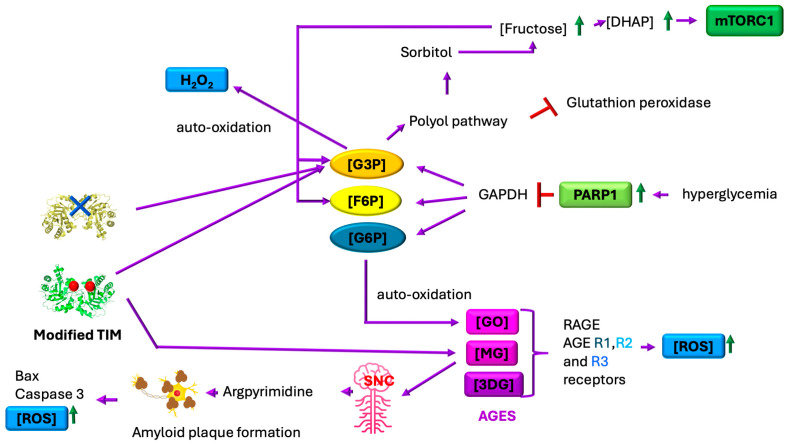
Consequences of oxidative/nitrative stress. In a state of hyperglycemia, poly (ADP-ribose) polymerase 1 (PARP1) represses the expression of glyceraldehyde-3-phosphate dehydrogenase (GAPDH), leading to the accumulation of glyceraldehyde 3-phosphate (G3P), fructose 6-phosphate (F6P), and glucose 6-phosphate (G6P). The auto-oxidation of G6P results in the formation of glyoxal (GO). GO, methylglyoxal (MG), and 3-deoxyglucosone (3DG) are advanced glycation end products (AGEs). When these AGEs bind to receptors such as RAGE or AGE R1, R2, and R3, they promote the formation of ROSs and reactive nitrogen species (NOSs). When TIM is absent (due to the repression of its expression by certain miRNAs during hyperglycemia) or is non-functional due to post-translational modifications, the levels of G3P and dihydroxyacetone phosphate (DHAP) increase. This activates the polyol pathway, consuming NADPH, impairing glutathione peroxidase activity, and worsening oxidative stress. Accumulation of fructose produces more DHAP, thus activating mTORC1 (an abundant DHAP level indicates abundant glucose availability, which cells interpret as a permissive signal for growth), which suppresses autophagy and promotes pyroptosis (inflammatory cell death) and ROS production. Meanwhile, G3P auto-oxidation generates hydrogen peroxide, further contributing to the oxidative environment.

Under diabetic hyperglycemic conditions, excessive ROS generation becomes intrinsically linked to dysregulated glucose and lipid metabolism. In patients with type 2 diabetes, basal intracellular ROSs are increased 3.4 ± 1.4-fold when compared with those in control subjects [6,73]. This oxidative overload disrupts insulin signaling pathways, leading to systemic insulin resistance. Chronic hyperglycemia creates a vicious cycle by suppressing both enzymatic (e.g., superoxide dismutase, catalase) and non-enzymatic antioxidant defenses across multiple tissues, resulting in uncontrolled ROS accumulation that further perpetuates metabolic dysfunction (in fact, the content of antioxidant enzymes in β-cells is 10- to 20-fold lower than in cells of the liver, kidneys, heart, brain, and other organs) [4,6] (Figure 8).

When TIM undergoes modification by RNSs and ROSs, its enzymatic activity becomes significantly impaired. This inactivation, coupled with the pathological accumulation of G3P and DHAP, drives the formation of MG. In the central nervous system (CNS), MG readily reacts with neuronal proteins, including TIM, catalyzing the formation of arginine-derived advanced glycation end-products (AGEs) such as argpyrimidine. These protein modifications promote amyloid plaque deposition, which subsequently (1) generates additional ROSs through oxidative chain reactions and (2) activates pro-apoptotic protein cascades, ultimately contributing to neurodegenerative pathology.

Under hyperglycemic conditions, glucose oxidation generates reactive oxygen species (ROSs) that induce DNA damage, activating the DNA repair enzyme poly (ADP-ribose) polymerase 1 (PARP1). PARP1 activation subsequently inhibits glyceraldehyde-3-phosphate dehydrogenase (GAPDH) activity, leading to the accumulation of glycolytic intermediates, including G3P, fructose-6-phosphate (F6P), and glucose-6-phosphate (G6P). Concurrently, protein-kinase-mediated pathways elevate G3P levels while stimulating the hexosamine and polyol pathways, further increasing F6P concentrations. This metabolic disruption drives three major pathological cascades: (1) formation of AGEs via reactions between reducing sugars (G6P/F6P) and proteins, generating RCSs such as MG, glyoxal (GO), and 3-deoxyglucosone (3DG); (2) ROS amplification through G3P auto-oxidation (producing H_2_O_2_) and G6P auto-oxidation (yielding GO); and (3) TIM enzyme dysfunction via oxidative-stress-induced non-enzymatic dephosphorylation (promoting MG formation) and nitrosative-stress-mediated nitration at Y164/Y208, which destabilizes the catalytic loop. This nitration reduces TIM activity ~15-fold by exposing the active site to water influx, further accelerating MG production. These interconnected pathways establish a self-perpetuating cycle of metabolic dysfunction and oxidative damage in diabetes [4,6,16,36,73,74,75] (Figure 1, Figure 2 and Figure 8).

MG exhibits broad reactivity, modifying diverse biomolecules including amino acids, nucleic acids, and proteins. Its primary targets are arginine residues, which it converts into argpyrimidine—a modification that disrupts neurotransmitter regulation by promoting excessive dopamine and serotonin release, potentially leading to central nervous system overstimulation. MG measurements in a TIM-deficient family revealed that the most severely affected individual exhibited a 59% increase in MG formation compared with controls, while diabetes mellitus patients showed an increase in MG production of approximately 54% [30,76,77].

Furthermore, argpyrimidine formation exacerbates neurodegenerative processes by accelerating amyloid-β plaque deposition and destabilizing mitochondrial membrane potential, which promotes the accumulation of pro-apoptotic factors (Bax and caspase-3) [76] (Figure 8). Beyond neurological effects, MG directly impairs insulin signaling in β-cells (INS-1E) and L6 myocytes by suppressing the IRS/PI3K/AKT pathway—an inhibition mechanism independent of oxidative stress. Clinically, elevated MG levels in diabetes drive endothelial dysfunction, contributing to microvascular complications including retinopathy, nephropathy, delayed wound healing, and microangiopathy [78].

The polyol pathway becomes pathologically activated during hyperglycemia, with aldose reductase hyperactivation driving excessive sorbitol production while depleting NADPH stores. This NADPH reduction impairs glutathione peroxidase activity, critically weakening cellular antioxidant defenses through glutathione depletion. The accumulated sorbitol is subsequently converted into fructose by sorbitol dehydrogenase, generating G3P and DHAP as byproducts. These metabolites, combined with NADH/NAD^+^ imbalance and ROS-induced TIM dysfunction, create conditions favoring the autoformation of MG. The resulting MG accumulation further exacerbates oxidative stress through two key mechanisms: activation of protein kinase C (PKC) pathways and stimulation of de novo diacylglycerol synthesis, establishing a self-perpetuating cycle of metabolic dysfunction [4,6] (Figure 8). In pancreatic β-cells, this oxidative damage manifests structurally through mitochondrial deformation and functionally via disintegration of K ATP, ultimately impairing both insulin secretion quantity and quality [4] (Figure 4). The cumulative effect represents a feedforward mechanism where hyperglycemia-induced oxidative stress both causes and results from progressive β-cell failure.

## 7. TIM and the Fluidity of Cell Membranes

Cell membranes are fundamental in modulating cellular contact and communication, as well as in the activity of enzymes that are part of them or interact with them to perform their functions; thus, their fluidity and composition are of utmost importance [79,80,81]. Changes in the quality and quantity of circulating fatty acids have a significant impact on the fluidity of cell membranes, particularly in erythrocytes, where this fluidity depends on the composition of available phospholipids (see Figure 2 and Figure 5) [80]. Numerous studies have found that the membranes of diabetic patients exhibit an excess of cholesterol and sphingomyelin in their composition, as well as an abundance of saturated fatty acids [80]. Erythrocytes in diabetic patients are abnormally more rigid than those of healthy individuals. This decrease in membrane fluidity has been observed in other cell types, such as ileal enterocytes of the intestinal brush border, sarcolemma of cardiac myocytes, leukocytes, synaptic vesicles in the cerebral cortex, and platelets [23,80]. Changes in the viscoelastic properties of erythrocyte membranes promote cardiovascular risk—a comorbidity associated with diabetes—making erythrocyte membrane fluidity a biomarker related to cardiovascular risk in patients with type 2 diabetes [81].

Particularly, membranes are susceptible to damage by ROSs and constant hyperglycemia. Erythrocytes are often used as models in the study of oxidative stress due to their simplicity and high availability. Human erythrocytes possess endogenous mechanisms to counteract damage associated with ROSs, such as catalase and superoxide dismutase (SOD); however, when levels of oxidative species are exceedingly high, oxidation of polyunsaturated fatty acids—the main components of the membrane—is promoted [79]. Insulin is an important regulator of membrane fluidity, as its signal(s) in the liver activate(s) fatty acid desaturases. Additionally, insulin activity promotes the induction of the GLUT4 transporter in plasma cells, as well as in the membranes of adipocytes and muscle cells. Another crucial factor that can affect the expression of membrane GLUT4 is the fluidity of the membrane itself; if there is a decrease in fluidity, expression halts, exacerbating hyperglycemia. Chronic low membrane fluidity reduces the expression of GLUT4 on the membrane, impairs insulin secretion and signaling, and leads to the stiffening of blood vessels [80].

TIM is the only glycolytic enzyme that also participates in fatty acid synthesis (Figure 2), and, as observed, TIM expression is affected in diabetic patients [3,25]. Although no direct studies exist on how TIM affects membrane fluidity in diabetes, it has been noted that patients with TIM deficiency exhibit fragility in erythrocytes. In 1997, Hollán [25] and collaborators reported a case where two twin brothers with TIM deficiency displayed differential neurodegeneration symptoms. Upon evaluating the lipid composition of the erythrocyte plasma membranes of both brothers, no significant difference was found between them or with healthy populations; however, a variation in the proportion of certain phospholipids was observed between the brothers and healthy subjects. This variation produces a change in membrane fluidity, directly affecting the function of some enzymes such as acetylcholinesterase and Ca^2+^ ATPase [25].

Additionally, previous studies in *Saccharomyces cerevisiae* have shown that the accumulation of DHAP and G3P due to the absence of TIM activity directly affects the de novo synthesis of inositol by inhibiting myo-inositol 3-phosphate [55]. This is significant because myoinositol, also known as cyclohexanehexol or inositol, is a carbohydrate with six hydroxyl groups (which belongs to a family of nine stereoisomers, considered non-essential since they are produced from glucose) that is part of cell membranes. All cells (animal, plant, bacterial, and fungal) contain inositol phospholipids in their membranes, either in free form or bound as phospholipids or inositol phosphates. Inositol also serves as the structural base for many hormonal second messengers, such as inositol triphosphate and inositol bisphosphate (IP3/IP2), generated from phosphatidylinositol 4,5-biphosphate (PIP2), as well as the inositol glycans that are linked to insulin signaling [56]. Therefore, changes in TIM activity also affect the composition of lipid membranes, as well as the signaling of insulin activity (Figure 2 and Figure 5).

Figure 2 and Figure 8 demonstrate that DHAP accumulation imbalance drives advanced glycation end-product (AGE) formation and disrupts phospholipid/triacylglycerol synthesis. Concomitant uncoupling of the pentose phosphate pathway—due to altered G3P availability—reduces NADPH production, impairing lipid synthesis. These observations demonstrate that TIM plays an important role in maintaining optimal viscoelastic properties of membranes, but this has barely been studied, except in patients with TIM deficiency. Research in this area could reveal new mechanisms of how metabolic diseases, such as diabetes, generally develop vascular problems. In animal models of TIM deficiency, many disease-associated alleles persist only heterozygously, as null alleles are typically lethal. These models recapitulate human symptomatology. Critically, all TIM impairment scenarios elevate DHAP, inducing oxidative stress via reactive oxygen/nitrogen species (ROS/RNS) accumulation [82,83,84,85,86,87,88,89].

## 8. Discussion

Diabetes is a complex metabolic disorder involving insulin deficiency and broader protein dysregulation that worsens over time. A key example is TIM, which connects energy and redox metabolism; its dysfunction causes severe metabolic disruptions, as seen in TIM deficiency. MicroRNAs such as miR-193b-3p serve as important disease markers, with elevated levels during hyperglycemia and insulin resistance making them potential prediabetes indicators. This miRNA directly targets TIM, reducing its expression, and it additionally suppresses GLUT2 transporters to impair glucose uptake. Interestingly, miR-193b-3p also affects the PGC-1α pathway in Parkinson’s disease, suggesting broader metabolic connections across disorders.

K ATP channels act as cellular energy sensors, opening during low glucose levels to maintain membrane hyperpolarization and block insulin release. When glucose enters and is metabolized, rising ATP levels close these channels, triggering membrane depolarization, calcium influx, and insulin secretion. However, recent findings show that K ATP channel subunits directly interact with glycolytic enzymes such as TIM, which independently modulates channel gating and insulin release. This explains how TIM deficiency, such as in diabetic patients with elevated miR-193b-3p suppressing TIM expression, disrupts normal insulin secretion regardless of energy status.

miR-1285-3p is dysregulated in both diabetes and pancreatic cancer patients. This miRNA also regulates TIM expression in Ovine Sertoli cells. Since TIM critically participates in male reproductive development, the suppression by this miRNA could impair fertility. This mechanism warrants further investigation, especially given its potential clinical relevance.

The mTOR pathway regulates critical cellular processes, exhibiting dual roles in diabetes by both promoting β-cell proliferation (compensating for insulin resistance) and contributing to tissue damage (e.g., kidney dysfunction and metabolic immune dysregulation). In breast cancer, TIM and CDCA5 activate mTORC1/PI3K/AKT signaling, suppressing apoptosis while enhancing glycolysis through GLUT1/4 upregulation and HIF1α/c-Myc-mediated glycolytic enzyme synthesis [36,57,60]. Although TIM-sororin’s role in normal mTORC1 activation remains unconfirmed, diabetic kidney disease demonstrates DHAP-driven mTORC1/ROS pathway activation that promotes podocyte pyroptosis, potentially mitigated by reducing DHAP levels [70]. Notably, TIM’s dopaminylation at Q65 shifts its activity toward G3P production, redirecting metabolism to prevent lipid peroxidation and ferroptosis and, thereby, supporting lung regeneration [42]. Under hyperglycemia, disrupted TIM expression impairs its ability to convert accumulating DHAP into G3P [3].

Under physiological conditions, ROSs function as signaling molecules, but diabetic redox imbalance disrupts this regulation, impairing insulin secretion and other processes. TIM is particularly vulnerable to ROS-/RNS-mediated modifications, including the nitration of Y164/Y208, which reduces its catalytic efficiency. This leads to DHAP/GA3P imbalance and methylglyoxal (MG) accumulation—a potent inhibitor of insulin secretion via IRS/PI3K/AKT pathway suppression in β-cells. Concurrent ROS/RNS activity depletes glutathione peroxidase, exacerbating DHAP/G3P accumulation. G3P auto-oxidizes to H_2_O_2_, further amplifying oxidative stress, while mitochondrial ROSs disrupt K ATP channels, compounding insulin secretion defects. Notably, accumulated DHAP also activates mTOR-driven pyroptosis, creating a vicious cycle of metabolic dysfunction.

Diabetes alters membrane composition across cell types, increasing rigidity and impairing membrane-associated protein function (including insulin secretion). TIM plays a crucial role in this process by regulating metabolites that influence lipid metabolism; its products connect to triacylglycerol synthesis, lipolysis, and glycolipid production via DHAP; glycolytic citrate (precursor to acetyl-CoA) supports phospholipid biosynthesis. While no direct studies confirm TIM’s membrane effects, TIM-deficient patients show altered membrane fluidity due to fatty acid imbalances, which disrupt membrane enzyme function and may contribute to neurodegeneration. These findings suggest that diabetes-induced TIM dysregulation could indirectly modify membrane properties through its metabolic network.

TIM’s essential metabolic role excludes its use as a direct therapeutic target. Inhibition risks toxic accumulation of DHAP, MG, and ROSs, while many of its moonlighting functions remain poorly understood. For instance, proposed TIM inhibitors for cancer (targeting the Warburg effect) could systemically harm healthy cells while tumors develop resistance. This exemplifies why single-target approaches such as conventional insulin therapy often fail in complex metabolic diseases. Instead, we advocate for system-level strategies that address the interconnected pathophysiology of diabetes and related disorders.

## 9. Conclusions

At present, the management of diabetes primarily focuses on enhancing glucose uptake through improved insulin delivery. However, as demonstrated by the review in this article, the development of the disease results from dysfunctions in multiple metabolic processes and various molecules that ultimately affect insulin secretion. Currently, multiple pharmacological therapies are available to enhance glucose release; however, it is now recognized that physical activity can independently resolve many of the aforementioned metabolic dysfunctions. For instance, light or moderate-intensity exercise improves glycemic control when performed intensely, increasing glucose uptake in muscle (lasting 2–48 h post-exercise) and when practiced routinely (where the effect of 1 h of exercise can extend for up to 3 days afterwards) through the reduction in glycated hemoglobin and blood glucose levels both in fasting and postprandial states. Regular aerobic or anaerobic physical activity can prevent both microvascular and macrovascular complications of diabetes mellitus by attenuating chronic inflammation associated with insulin resistance and chronic hyperglycemia. Additionally, aerobic exercise may decrease the progression or prevent the onset of peripheral neuropathy, among other benefits. In general, exercise prescription for patients with diabetes should be evaluated by a health specialist and will depend on the presence of acute or chronic complications [90].

Given the high incidence of this condition, a crucial approach would be to focus on and prioritize the correction of these metabolic dysfunctions to facilitate the restoration of homeostasis, with the aid of pharmacological therapies alongside personalized physical activity regimens for the patient.

## Data Availability

No new data were created or analyzed in this study.

## Abbreviations

The following abbreviations are used in this manuscript:

3DG	3-deoxyglucosone;
ABC	ATP-binding cassette;
AGE	advanced glycation end product;
AKT	protein kinase B;
AP1	activator protein 1;
CDCA5	cell division cycle associated 5 or sororin;
c-Myc	MYC proto-oncogene;
DHAP	dihydroxyacetone phosphate;
F6P	fructose 6-phosphate;
FOXO1	forkhead box protein O1;
G3P	glyceraldehyde 3-phosphate;
G6P	glucose 6-phosphate;
GAPDH	glyceraldehyde 3-phosphate dehydrogenase;
GATOR complex	regulator of the Rag GTPases;
GLUT1	glucose transporter 1;
GLUT2	glucose transporter 2;
GLUT4	glucose transporter 4;
GO	glyoxal;
Hep G2	human liver cancer cell line;
HIF1α	hypoxia inducible factor 1 alpha;
HIPPO	Salvador-Warts-Hippo (SWH) pathway;
IRS	insulin receptor substrate;
K ATP	ATP-sensitive potassium channels;
MG	methylglyoxal;
miRNA	microRNA;
mTOR	mammalian target of rapamycin;
mTORC1	mammalian target of rapamycin complex 1;
mTORC2	mammalian target of rapamycin complex 2;
NLRP3	nucleotide-binding domain leucine-rich repeat family pyrin domain containing 3;
NOX	NADPH oxidase;
PARP1	poly(ADP-ribose) polymerase 1;
PI3K	phosphoinositide 3-kinase;
PK	pyruvate kinase;
PPARGC1A	peroxisome proliferator receptor gamma coactivator 1 alpha;
RAGE	receptor for advanced glycation end products;
RCS	reactive dicarbonyl molecules;
RNS	reactive nitrogen species;
ROS	reactive oxygen species;
SOD	superoxide dismutase;
SOS2	Son of Sevenless 2;
SUR1	sulfonylurea receptor 1;
SUR2	sulfonylurea receptor 2;
TIM	triosephosphate isomerase;
UTR	untranslated region;
YAP/YAP1	yes associated protein 1;
YWHAZ/14-3-3ζ	gene coding for tyrosine 3-monooxygenase/tryptophan 5-monooxygenase activation protein zeta/14-3-3 protein zeta.

## References

[1] Cloete L. (2022). Diabetes mellitus: An overview of the types, symptoms, complications and management. Nurs. Stand..

[2] Defeudis G., Mazzilli R., Tenuta M., Rossini G., Zamponi V., Olana S., Faggiano A., Pozzilli P., Isidori A.M., Gianfrilli D. (2022). Erectile dysfunction and diabetes: A melting pot of circumstances and treatments. Diabetes Metab. Res..

[3] Hu H., Zhao M., Li Z., Nie H., He J., Chen Z., Yuan J., Guo H., Zhang X., Yang H. (2022). Plasma miR-193b-3p is elevated in type 2 diabetes and could impair glucose metabolism. Front. Endocrinol..

[4] Ighodaro O.M. (2018). Molecular pathways associated with oxidative stress in diabetes mellitus. Biomed. Pharmacother..

[5] Luo A., Xie Z., Wang Y., Wang X., Li S., Yan J., Zhan G., Zhou Z., Li S. (2022). Type 2 diabetes mellitus-associated cognitive dysfunction: Advances in potential mechanisms and therapies. Neurosci. Biobehav. Rev..

[6] Darenskaya M.A., Kolesnikova L.I., Kolesnikov S.I. (2021). Oxidative stress: Pathogenetic role in diabetes mellitus and its complications and therapeutic approaches to correction. Bull. Exp. Biol. Med..

[7] Agrawal R., Reno C.M., Sharma S., Christensen C., Huang Y., Fisher S.J. (2021). Insulin action in the brain regulates both central and peripheral functions. Am. J. Physiol. Endocrinol. Metab..

[8] Formiga F., Pérez-Maraver M. (2014). Diabetes mellitus tipo 3. ¿El renacer de la insulina inhalada?. Endocrinol. Nutr..

[9] Janoutová J., Machaczka O., Zatloukalová A., Janout V. (2022). Is Alzheimer’s disease a type 3 diabetes? A review. Cent. Eur. J. Public Health.

[10] (2024). Centers for Disease Control and Prevention. https://www.cdc.gov/diabetes/prevention-type-2/index.html.

[11] Medina-Pérez E.A., Sánchez-Reyes A., Hernández-Peredo A.R., Martínez-López M.A., Jiménez-Flores C.N., Serrano-Ortiz I., Maqueda-Pineda A.V., Islas-Cruz D.N., Cruz-González M. (2017). Diabetes gestacional. Diagnóstico y tratamiento en el primer nivel de atención. Med. Interna México.

[12] Grizzanti J., Moritz W.R., Pait M.C., Stanley M., Kaye S.D., Carroll C.M., Constantino N.J., Deitelzweig L.J., Snipes J.A., Kellar D. (2023). KATP channels are necessary for glucose-dependent increases in amyloid-β and Alzheimer’s disease–related pathology. JCI Insight.

[13] Ohtsuki S. (2024). Insulin receptor at the blood–brain barrier: Transport and signaling. Vitam. Horm..

[14] Mesarosova L., Scheper M., Iyer A., Anink J.J., Mills J.D., Aronica E. (2024). miR-193b-3p/PGC-1α pathway regulates an insulin dependent anti-inflammatory response in Parkinson’s disease. Neurobiol. Dis..

[15] Hernández-Alcántara G., Garza-Ramos G., Hernández G.M., Gómez-Puyou A., Perez-Montfort R. (2002). Catalysis and stability of triosephosphate isomerase from *Trypanosoma brucei* with different residues at position 14 of the dimer interface. Characterization of a catalytically competent monomeric enzyme. Biochemistry.

[16] Katebi A.R., Jernigan R.L. (2014). The critical role of the loops of triosephosphate isomerase for its oligomerization, dynamics, and functionality. Prot. Sci..

[17] Knowles J.R. (1991). Enzyme catalysis: Not different, just better. Nature.

[18] Wierenga R.K., Kapetaniou E.G., Venkatesan R. (2010). Triosephosphate isomerase: A highly evolved biocatalyst. Cell Mol. Life Sci..

[19] Bar-Even A., Flamholz A., Noor E., Milo R. (2012). Rethinking glycolysis: On the biochemical logic of metabolic pathways. Nat. Chem. Biol..

[20] Helliwell J.R. (2021). Triosephosphate isomerase: The perfect enzyme, but how does it work?. IUCrJ.

[21] Karg E., Németh I., Horányi M., Pintér S., Vécsei L., Hollán S. (2000). Diminished blood levels of reduced glutathione and α-tocopherol in two triosephosphate isomerase-deficient brothers. Blood Cells Mol. Dis..

[22] Sun T., Tan L., Liu M., Zeng L., Zhao K., Cai Z., Sun S., Li Z., Liu R. (2023). Tilianin improves cognition in a vascular dementia rodent model by targeting miR-193b-3p/CaM- and miR-152-3p/CaMKIIα-mediated inflammatory and apoptotic pathways. Front. Immunol..

[23] Orosz F., Oláh J., Ovádi J. (2009). Triosephosphate isomerase deficiency: New insights into an enigmatic disease. Biochim. Biophys. Acta Mol. Basis Dis..

[24] Asakawa J., Mohrenweiser H.W. (1982). Characterization of two new electrophoretic variants of human triosephosphate isomerase: Stability, kinetic, and immunological properties. Biochem. Genet..

[25] Perry B.A., Mohrenweiser H.W. (1992). Human triosephosphate isomerase: Substitution of Arg for Gly at position 122 in a thermolabile electromorph variant, TPI-Manchester. Hum. Genet..

[26] Watanabe M., Zingg B.C., Mohrenweiser H.W. (1996). Molecular analysis of a series of alleles in humans with reduced activity at the triosephosphate isomerase locus. Am. J. Hum. Genet..

[27] Hollán S., Magócsi M., Fodor E., Horányi M., Harsányi V., Farkas T. (1997). Search for the pathogenesis of the differing phenotype in two compound heterozygote Hungarian brothers with the same genotypic triosephosphate isomerase deficiency. Proc. Natl. Acad. Sci. USA.

[28] Arya R., Lalloz M.R.A., Bellingham A.J., Layton D.M. (1997). Evidence for founder effect of the glu104asp substitution and identification of new mutations in triosephosphate isomerase deficiency. Hum. Mutat..

[29] Ationu A., Humphries A., Lalloz M.R.A., Arya R., Wild B., Warrilow J., Morgan J., Bellingham A.J., Layton D.M. (1999). Reversal of metabolic block in glycolysis by enzyme replacement in triosephosphate isomerase–deficient cells. Blood.

[30] Ahmed N., Battah S., Karachalias N., Babaei-Jadidi R., Horányi M., Baróti K., Hollán S., Thornalley P.J. (2003). Increased formation of methylglyoxal and protein glycation, oxidation and nitrosation in triosephosphate isomerase deficiency. Biochim. Biophys. Acta Mol. Basis Dis..

[31] Oláh J., Orosz F., Puskás L.G., Hackler L., Horányi M., Polgár L., Hollán S., Ovadi J. (2005). Triosephosphate isomerase deficiency: Consequences of an inherited mutation at mRNA, protein and metabolic levels. Biochem. J..

[32] Ralser M., Heeren G., Breitenbach M., Lehrach H., Krobitsch S. (2006). Triose phosphate isomerase deficiency is caused by altered dimerization–not catalytic inactivity–of the mutant enzymes. PLoS ONE.

[33] Rodríguez-Almazán C., Arreola R., Rodríguez-Larrea D., Aguirre-López B., De Gómez-Puyou M.T., Perez-Montfort R., Costas M., Gómez-Puyou A., Torres-Larios A. (2008). Structural basis of human triosephosphate isomerase deficiency. J. Biol. Chem..

[34] Fermo E., Bianchi P., Vercellati C., Rees D.C., Marcello A.P., Barcellini W., Zanella A. (2010). Triose phosphate isomerase deficiency associated with two novel mutations in TPI gene. Eur. J. Haematol..

[35] Serdaroglu G., Aydinok Y., Yilmaz S., Manco L., Özer E. (2011). Triosephosphate isomerase deficiency: A patient with Val231Met mutation. Pediatr. Neurol..

[36] Roland B.P., Amrich C.G., Kammerer C.J., Stuchul K.A., Larsen S.B., Rode S., Aslam A.A., Heroux A., Wetzel R., VanDemark A.P. (2015). Triosephosphate isomerase I170V alters catalytic site, enhances stability and induces pathology in a Drosophila model of TPI deficiency. Biochim. Biophys. Acta Mol. Basis Dis..

[37] Cabrera N., Torres-Larios A., García-Torres I., Enríquez-Flores S., Perez-Montfort R. (2018). Differential effects on enzyme stability and kinetic parameters of mutants related to human triosephosphate isomerase deficiency. Biochim. Biophys. Acta Gen. Subj..

[38] Jin X., Wang D., Lei M., Guo Y., Cui Y., Chen F., Sun W., Chen X. (2022). TPI1 activates the PI3K/AKT/mTOR signaling pathway to induce breast cancer progression by stabilizing CDCA5. J. Transl. Med..

[39] Duan Y., Li J., Wang F., Wei J., Yang Z., Sun M., Liu J., Wen M., Huang W., Chen Z. (2021). Protein modifications throughout the lung cancer proteome unravel the cancer-specific regulation of glycolysis. Cell Rep..

[40] De La Mora-de La Mora I., Torres-Larios A., Enríquez-Flores S., Méndez S.T., Castillo-Villanueva A., Gómez-Manzo S., López-Velázquez G., Marcial-Quino J., Torres-Arroyo A., García-Torres I. (2015). Structural effects of protein aging: Terminal marking by deamidation in human triosephosphate isomerase. PLoS ONE.

[41] Hipkiss A.R. (2016). Activity-induced deamidation of triose-phosphate isomerase may explain the deleterious effects of excessive glucose consumption. Int. J. Diabetes Clin. Res..

[42] Mo C., Li H., Yan M., Xu S., Wu J., Li J., Yang X., Li Y., Yang J., Su X. (2024). Dopaminylation of endothelial TPI1 suppresses ferroptotic angiocrine signals to promote lung regeneration over fibrosis. Cell Metab..

[43] Rodríguez-Bolaños M., Perez-Montfort R. (2019). Medical and veterinary importance of the moonlighting functions of triosephosphate isomerase. Curr. Protein Pept. Sci..

[44] Myers T.D., Palladino M.J. (2023). Newly discovered roles of triosephosphate isomerase including functions within the nucleus. Mol. Med..

[45] Suksangrat T., Phannasil P., Jitrapakde S., Guest P. (2019). miRNA regulation of glucose and lipid metabolism in relation to diabetes and non-alcoholic fatty liver disease. Reviews on Biomarker Studies of Metabolic and Metabolism-Related Disorders.

[46] Mollet I.G., Macedo M.P. (2023). Pre-diabetes-linked miRNA miR-193b-3p targets PPARGC1A, disrupts metabolic gene expression profile and increases lipid accumulation in hepatocytes: Relevance for MAFLD. Int. J. Mol. Sci..

[47] Kolokotronis T., Majchrzak-Stiller B., Buchholz M., Mense V., Strotmann J., Peters I., Skrzypczyk L., Liffers S.T., Menkene L.M., Wagner M. (2024). Differential miRNA and protein expression reveals miR-1285, its targets TGM2 and CDH-1, as well as CD166 and S100A13 as potential new biomarkers in patients with diabetes mellitus and pancreatic adenocarcinoma. Cancers.

[48] Zhang C., He J., Xiong D., Mei Y., Zhu Y., Deng P., Duan Y. (2025). Effect of miR-1285-3p as a diagnostic biomarker for chronic heart failure on vascular endothelial cells. J. Cardiothorac. Surg..

[49] An X., Li T., Chen N., Wang H., Su M., Shi H., Duan X., Ma Y. (2022). miR-1285-3p targets TPI1 to regulate the glycolysis metabolism signaling pathway of Tibetan sheep Sertoli cells. PLoS ONE.

[50] Olveira F.P., Martins A.D., Moreire A.C., Cheng C.Y., Alves M.G. (2014). The Warburg effect revisited—Lesson from the Sertoli cell. Med. Res. Rev..

[51] Galardo M.N., Gorga A., Merlo J.P., Regueira M., Pellizzari E.H., Cigorraga S.B., Riera M.F., Meroni S.B. (2017). Participation of HIFs in the regulation of Sertoli cell lactate production. Biochimie.

[52] Hermo L., Oliveira R.L., Smith C.E., Au C.E., Bergeron J.J.M. (2019). Dark side of epididymis: Tails of sperm maturation. Andrology.

[53] Auer J., Camoin L., Courtot A., Hotellier F., De Almeida M. (2004). Evidence that P36, a human sperm acrosomal antigen involved in the fertilization process is triosephosphate isomerase. Mol. Reprod. Dev..

[54] Byrne K., Leahy T., McCulloch R., Colgrave M.L., Holland M.K. (2012). Comprehensive mapping of the bull sperm surface proteome. Proteom. Syst. Biol..

[55] Takei G.L., Miyashiro D., Mukai C., Okuno M. (2014). Glycolisis plays an important role in energy transfer from the base to the distal end flagellum in mouse sperm. J. Exp. Biol..

[56] Yang L., Shen J., Chen J., Li W., Xie X. (2019). Reduced glycolysis contributed to inhibition of testis spermatogenesis in rats after chronic methamphetamine exposure. Med. Sci. Monit..

[57] Auer J., Senechal H., Desvaux F.X., Albert M., De Almeida M. (2000). Isolation and isolation of two sperm membrane proteins recognized by sperm-associated antibodies in infertile men. Mol. Reprod. Dev..

[58] Zangbar M.S., Keshtgar S., Zolghadri J., Gharesi-Fard B. (2016). Antisperm protein targets in azoospermia men. J. Hum. Reprod. Sci..

[59] Daniel B., Livne A., Cohen G., Kahremany S., Sasson S. (2021). Endothelial cell–derived triosephosphate isomerase attenuates insulin secretion from pancreatic beta cells of male rats. Endocrinology.

[60] Martin G.M., Sung M.W., Yang Z., Innes L.M., Kandasamy B., David L.L., Yoshioka C., Shyng S.L. (2019). Mechanism of pharmacochaperoning in a mammalian K_ATP_ channel revealed by cryo-EM. eLife.

[61] Dhar-Chowdhury P., Harrell M.D., Han S.Y., Jankowska D., Parachuru L., Morrissey A., Srivastava S., Liu W., Malester B., Yoshida H. (2005). The glycolytic enzymes, glyceraldehyde-3-phosphate dehydrogenase, triose-phosphate isomerase, and pyruvate kinase are components of the K_ATP_ channel macromolecular complex and regulate its junction. J. Biol. Chem..

[62] Shi Y., Vaden D.L., Ju S., Ding D., Geiger J.H., Greenberg M.L. (2005). Genetic perturbation of glycolysis results in inhibition of de novo inositol biosynthesis. J. Biol. Chem..

[63] DiNicolantonio J.J., O’Keefe J.H. (2022). Myo-inositol for insulin resistance, metabolic syndrome, polycystic ovary syndrome and gestational diabetes. Open Heart.

[64] Laplante M., Sabatini D.M. (2009). mTOR signaling at a glance. J. Cell Sci..

[65] Butterfield D.A., Halliwell B. (2019). Oxidative stress, dysfunctional glucose metabolism and Alzheimer disease. Nat. Rev. Neurosci..

[66] Chen T., Huang Z., Tian Y., Wang H., Ouyang P., Chen H., Wu L., Lin B., He R. (2017). Role of triosephosphate isomerase and downstream functional genes on gastric cancer. Oncol. Rep..

[67] Shen A., Liu L., Chen H., Qi F., Huang Y., Lin J., Sferra T.J., Sankararaman S., Wei L., Chu J. (2019). Cell division cycle associated 5 promotes colorectal cancer progression by activating the ERK signaling pathway. Oncogenesis.

[68] Orozco J.M., Krawczyk P.A., Scaria S.M., Cangelosi A.L., Chan S.H., Kunchok T., Lewis C.A., Sabatini D.M. (2020). Dihydroxyacetone phosphate signals glucose availability to mTORC1. Nat. Metab..

[69] Riboulet-Chavey A., Pierron A., Durand I., Murdaca J., Giudicelli J., Van Obberghen E. (2006). Methylglyoxal impairs the insulin signaling pathways independently of the formation of intracellular reactive oxygen species. Diabetes.

[70] Zhang Z., Hu H., Luo Q., Yang K., Zou Z., Shi M., Liang W. (2023). Dihydroxyacetone phosphate accumulation leads to podocyte pyroptosis in diabetic kidney disease. J. Cell. Mol. Med..

[71] Vergès B. (2018). mTOR and cardiovascular diseases: Diabetes mellitus. Transplantation.

[72] Tuo Y., Xiang M. (2019). mTOR: A double-edged sword for diabetes. J. Leukoc. Biol..

[73] Restaino R.M., Deo S.H., Parrish A.R., Fadel P.J., Padilla J. (2017). Increased monocyte-derived reactive oxygen species in type 2 diabetes: Role of endoplasmic reticulum stress. Exp. Physiol..

[74] Bellier J., Nokin M.J., Lardé E., Karoyan P., Peulen O., Castronovo V., Bellahcène A. (2019). Methylglyoxal, a potent inducer of AGEs, connects between diabetes and cancer. Diabetes Res. Clin. Pract..

[75] Gao W., Zhao J., Li H., Gao Z. (2017). Triosephosphate isomerase tyrosine nitration induced by heme–NaNO_2_–H_2_O_2_ or peroxynitrite: Effects of different natural phenolic compounds. J. Biochem. Mol. Toxicol..

[76] Tajes M., Eraso-Pichot A., Rubio-Moscardó F., Guivernau B., Bosch-Morató M., Valls-Comamala V., Muñoz F.J. (2014). Methylglyoxal reduces mitochondrial potential and activates Bax and caspase-3 in neurons: Implications for Alzheimer’s disease. Neurosci. Lett..

[77] Reyaz A., Alam S., Chandra K., Kohli S., Agarwal S. (2020). Methylglyoxal and soluble RAGE in type 2 diabetes mellitus: Association with oxidative stress. J. Diabetes Metab. Disord..

[78] Nigro C., Leone A., Raciti G., Longo M., Mirra P., Formisano P., Beguinot F., Miele C. (2017). Methylglyoxal-glyoxalase 1 balance: The root of vascular damage. Int. J. Mol. Sci..

[79] Margina D., Ilie M., Gradinaru D., Vladica M., Pencea C., Mitrea N., Katona E. (2009). Redox status parameters and PBMC membrane fluidity in diabetes mellitus. Clin. Exp. Med. J..

[80] Pilon M. (2016). Revisiting the membrane-centric view of diabetes. Lipids Health Dis..

[81] Bianchetti G., Cefalo C.M.A., Ferreri C., Sansone A., Vitale M., Serantoni C., Abeltino A., Mezza T., Ferraro P.M., De Spirito M. (2024). Erythrocyte membrane fluidity: A novel biomarker of residual cardiovascular risk in type 2 diabetes. Eur. J. Clin. Investig..

[82] Merkle S., Pretsch W. (1989). Characterization of triosephosphate isomerase mutants with reduced enzyme activity in *Mus musculus*. Genetics.

[83] Celotto A.M., Frank A.C., Seigle J.L., Palladino M.J. (2006). Drosophila model of human inherited triosephosphate isomerase deficiency glycolytic enzymopathy. Genetics.

[84] Hrizo S.L., Eicher S.L., Myers T.D., McGrath I., Wodrich A.P.K., Venkastesh H., Manjooran D., Swoger S., Gagnon K., Bruskim M. (2021). Identification of protein quality control regulators using a Drosophila model of TPI deficiency. Neurobiol. Dis..

[85] Stone A., Cujic O., Rowlett A., Aderhold S., Savage E., Graham B., Steinert J.R. (2023). Triose-phosphate isomerase deficiency is associated with a dysregulation of synaptic vesicle recycling in *Drosophila melanogaster*. Front. Synaptic Neurosci..

[86] Ginger J.P., Kreber R.A., Ganetzky B. (2006). Wasted away, a Drosophila mutation in triosephosphate isomerase, causes paralysis, neurodegeneration, and early death. Proc. Natl. Acad. Sci. USA.

[87] Ralser M., Nebel A., Kleindorp R., Krobitsch S., Lehrach H., Schreiber S., Reinhardt R., Timmermann B. (2008). Sequencing and genotypic analysis of the triosephosphate isomerase (TPI1) locus in a large sample of long-lived Germans. BMC Genet..

[88] Sun P., Li Y., Liu F., Wang L. (2024). Generation and analysis of TPI deficiency zebrafish model. Yi Chuan.

[89] Myers T., Ferguson C., Gliniak E., Homaniacs E.G., Palladino M.J. (2022). Murine model of triosephosphate isomerase deficiency with anemia and severe neuromuscular dysfunction. Curr. Res. Neurobiol..

[90] Nieto-Martínez R. (2010). Actividad física en la prevención y tratamiento de la diabetes. Rev. Venez. Endocrinol. Metab..

